# Efficacy of Serum Ferritin–Zinc Ratio for Predicting Advanced Liver Fibrosis in Patients with Autoimmune Hepatitis

**DOI:** 10.3390/jcm12134463

**Published:** 2023-07-03

**Authors:** Kei Moriya, Shinya Sato, Norihisa Nishimura, Hideto Kawaratani, Hiroaki Takaya, Kosuke Kaji, Tadashi Namisaki, Masakazu Uejima, Shinsaku Nagamatsu, Hideki Matsuo, Hitoshi Yoshiji

**Affiliations:** 1 Department of Gastroenterology, Nara Medical University, Kashihara 634-8521, Japan; shinyasato@naramed-u.ac.jp (S.S.); nishimuran@naramed-u.ac.jp (N.N.); kawara@naramed-u.ac.jp (H.K.); htky@naramed-u.ac.jp (H.T.); kajik@naramed-u.ac.jp (K.K.); tadashin@naramed-u.ac.jp (T.N.); m3m16d333@yahoo.co.jp (M.U.); joe.montana.no16@gmail.com (S.N.); h.matsuo@nara-hp.jp (H.M.); yoshijih@naramed-u.ac.jp (H.Y.); 2 Department of Gastroenterology, Nara Prefecture General Medical Center, 897-5, 2-Chome, Shichijo-Nishimachi, Nara 630-8581, Japan

**Keywords:** autoimmune hepatitis, zinc, ferritin, liver fibrosis, biomarker

## Abstract

**Background/Aims:** The search for noninvasive biomarkers that can efficiently estimate the extent of liver fibrosis progression is ongoing. Although Fibrosis-4 (FIB-4), the aspartate transaminase-to-platelet ratio index (APRI), and the Forns index have been reported as useful biomarkers, their investigation in autoimmune hepatitis (AIH) is limited. This study aimed to examine the usefulness of these serological indices and a newly developed index in predicting liver fibrosis progression in AIH. **Methods:** The study analyzed data from 190 patients diagnosed with AIH at our institution between 1990 and 2015. Their histological liver fibrosis progression and clinical long-term prognosis were evaluated retrospectively (cohort 1). In 90 patients, receiver operating characteristic (ROC) curves were compared to choose severe fibrosis cases with respect to existing indices (FIB-4, APRI, and Forns index) and the ferritin–zinc ratio (cohort 2). **Results:** In cohort 1, liver-related death and hepatocellular carcinoma rates were significantly higher in the severe (n = 27) than in the mild (n = 63) fibrosis group (*p* = 0.0001 and 0.0191, respectively). In cohort 2, liver-related death in the severe fibrosis group was significantly frequent (*p* = 0.0071), and their ferritin–zinc ratio was higher (median 2.41 vs. 0.62, *p* = 0.0011). ROC analyses were performed to compare the ability of the ferritin–zinc ratio, FIB-4, APRI, and the Forns index to predict severe and mild fibrosis. Accordingly, areas under the ROC were 0.732, 0.740, 0.721, and 0.729, respectively. **Conclusions:** The serum ferritin–zinc ratio can noninvasively predict liver fibrosis progression in AIH and be applied to predict long-term prognosis.

## 1. Introduction

The number of patients with autoimmune hepatitis (AIH), a chronic inflammatory liver disease with unknown etiology, is increasing [1]; Meanwhile, hepatitis B and C are well managed, and their rates gradually decreased because of the positive effects of universal vaccination or direct-acting antivirals [2,3]. The progression of fibrosis is an important factor for predicting long-term prognosis of patients with chronic liver diseases, including AIH [4,5]. Liver biopsy is regarded as the most reliable tool for assessing the degree of hepatic fibrosis; however, repeated biopsies are not advised because of invasiveness and serious complications [6]. Instead of liver biopsy, the use of other assessment methods such as biomarkers and radiological tools has been proposed. The efficacy of biomarkers such as Fibrosis-4 (FIB-4) [7], the aspartate transaminase-to-platelet ratio index (APRI) [8], and the Forns index [9] were well validated for chronic hepatitis C and cirrhosis; however, the prognostic usefulness of these indices for AIH has not been examined enough up to now. Efe et al. recently reported the efficacy of angiotensin-converting enzyme (ACE) as a fibrosis-predicting tool for patients with AIH [10]. Although convenient to use, it is not suitable for patients taking hypotensive drugs such as ACE inhibitors and angiotensin II receptor blockers. Radiological tools, such as Shear-wave^TM^, Fibro-scan^TM^, and Elastgraphy^TM^, are not universally prevalent in many medical facilities but are simple and convenient to use [11,12,13,14].

Iron is the largest mineral element in the human body, and 70% of iron is present in the hemoglobin of red blood cells. The remaining percentage is stored as ferritin and hemosiderin in the liver, spleen, and bone marrow. A meta-analysis reported that a high serum ferritin level is a risk factor of death in patients with decompensated cirrhosis [15].

Zinc is the second largest mineral element in the human body and has important anti-inflammatory, antioxidant, and anti-apoptotic effects [16]. Zinc also has a pivotal role in supporting the activity of collagen in synthesizing enzymes [17]. Because collagenase, a main protease for the degradation of bound collagens, is a zinc metalloenzyme, zinc deficiency decreases its activity and subsequently leads to liver fibrosis [18]. Recently, we have reported that not only serum fibrotic markers but also patients’ quality of life improved following zinc supplementation in patients with AIH [19,20].

In this study, we examined a potential role of the serum ferritin–zinc ratio for estimating hepatic fibrosis progression in patients with AIH and predicting their long-term prognosis.

## 2. Methods

### 2.1. Patients

**Figure 1:**
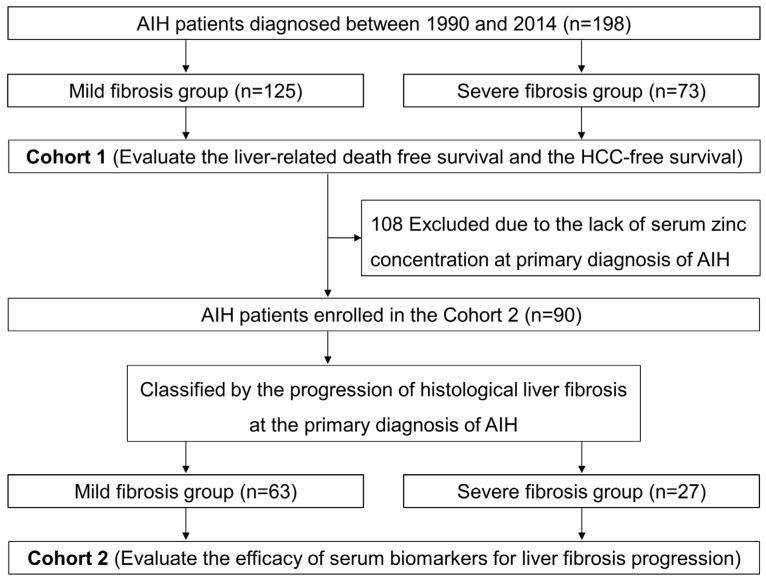
Flow chart of patients in this study. In cohort 1, liver-related death-free survival and HCC-free survival were evaluated in patients with AIH. In cohort 2, the efficacy of serum biomarkers for predicting long-term prognosis was evaluated in patients with AIH. AIH, autoimmune hepatitis; HCC, hepatocellular carcinoma.

A total of 198 consecutive patients with AIH who attended Nara Medical University Hospital between January 1990 and December 2015 were enrolled in cohort 1 for an observational clinical study (Figure 1). All of them were appropriately diagnosed based on the international AIH criteria suggested by the International Autoimmune Hepatitis Group [21] with histopathological findings, except for a few definite cases of liver cirrhosis. Patients who were infected with the hepatitis B virus (HBV), hepatitis C virus (HCV), or human immunodeficiency virus and had a history of excessive alcohol consumption were excluded. Of the 198 patients with AIH, 90 who had their laboratory results of serum zinc concentration at the primary diagnosis of AIH were also enrolled in cohort 2. In both cohorts, patients with AIH were divided into two groups based on each progression level of hepatic fibrosis at the time of primary diagnosis. Patients treated with zinc acetate, zinc sulfate, polaprezinc, or zinc-containing supplement at the time of primary histological diagnosis were also excluded.

### 2.2. Laboratory Assessments

During their first or second visit to our hospital, all patients underwent a routine laboratory examination, including a complete blood count, general biochemistry test, and coagulation test. Serum zinc and ferritin concentrations were measured by BML Inc. (Tokyo, Japan) using the atomic absorption spectrophotometry method and the latex agglutination method, respectively. Serological assessment of antinuclear antibody was performed according to a standard autoantibody testing procedure. 

### 2.3. Formula of Noninvasive Serum Tests

In this study, noninvasive serum tests for predicting liver fibrosis progression were as follows: FIB-4 = (age (years) × AST (U/L))/((PLT count (10^9^/L) × (ALT (U/L))^1/2^), APRI = (AST (U/L)/ULN of AST)/PLT count (10^9^/L) × 100, and Forns index = 7.811 − 3.131 × In (PLT count (10^9^/L)) + 0.781 × In (gamma-GT(U/L)) + 3.467 × In (age (years)) − 0.014 × (cholesterol (mg/dL)).

### 2.4. Histological Assessments

Liver tissue was biopsied by the ultrasonographically guided percutaneous puncture method on patient’s right lobe using a 16G or 18G needle, promptly after the patient was referred to our hospital. The choice of needle size was made depending on the investigator’s preference as well as its availability. Biopsy samples were stained with hematoxylin/eosin and Azan. Liver fibrosis was staged based on the METAVIR score (F0, no fibrosis; F1, portal fibrosis without septa; F2, portal fibrosis with few septa; F3, numerous septa without cirrhosis; and F4, cirrhosis) [22]. F0–F2 was considered no to mild fibrosis, whereas F3–F4 was considered severe. Histological evaluation was performed by two experienced pathologists.

### 2.5. HCC Assessments

Hepatocellular carcinoma (HCC) was clinically diagnosed according to the diagnostic algorithms for hepatocellular carcinoma in the clinical practice guidelines for hepatocellular carcinoma 2013 proposed by the Japan Society of Hepatology [23].

### 2.6. Statistical Analyses

Unrelated categorical variables were examined using Pearson’s chi-square test. Numerical variables were expressed as the median and quartiles. The Mann–Whitney U-test was used to evaluate the differences between two groups without a normal distribution. The correlation was assessed using Spearman’s rank correlation coefficients. The log-rank test was used to determine significant differences in Kaplan–Meier curves. Receiver operating characteristic (ROC) curves were compared to evaluate the predictive accuracy of different noninvasive serum tests. The area under the receiver operating characteristic (AUROC) curve was used to assess the predictive value of the variables. Differences between the AUROCs were evaluated using the z-test. Optimal cutoff values between mild and severe fibroses were identified at the maximum total sensitivity and specificity. *p* values of <0.05 were considered indicative of statistical significance. JMP version 14.3 (SAS Institute Inc., Cary, NC, USA) software was used for statistical analyses.

### 2.7. Ethical Issues

The Ethical Committee of Nara Medical University approved this study (Approval nos. 1116 and 15-003), which was conducted according to the ethical principles of the Japanese ethics guideline for life science and medical research involving human subjects (https://www.mhlw.go.jp/content/000769923.pdf. Accessed on 21 May 2023). This study was conducted according to the Declaration of Helsinki, and informed consent was obtained by an opt-out method.

## 3. Results

### 3.1. Long-Term Prognosis of Patients with AIH

**Figure 2:**
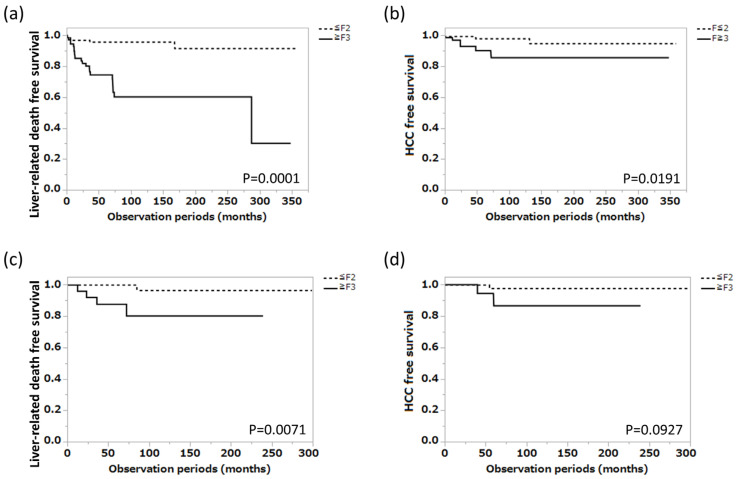
Relations between the degree of liver fibrosis progression and major clinical outcomes. (**a**) Liver-related death-free survival in the mild and severe liver fibrosis groups in cohort 1 (n = 198). (**b**) HCC-free survival in the mild and severe liver fibrosis groups in cohort 1 (n = 198). (**c**) Liver-related death-free survival in the mild and severe liver fibrosis groups in cohort 2 (n = 90). (**d**) HCC-free survival in the mild and severe liver fibrosis groups in cohort 2 (n = 90). HCC, hepatocellular carcinoma.

The clinical profile of the 198 patients with AIH enrolled in cohort 1 is shown in Table 1. The median age was 62.0 years, the male-to-female ratio was 33/165, and the liver fibrosis progression values were 76, 49, 40, and 33 for F1, F2, F3, and F4, respectively. The median serum alanine aminotransferase (ALT), total bilirubin, albumin, and IgG were 81.5 U/L, 1.1 mg/dL, 4.0 g/dL, and 2090 mg/dL, respectively. This patient group was divided into the mild fibrosis group (n = 125) and the severe fibrosis group (n = 73) based on histological liver fibrosis progression, and liver-related death-free survival in each group was compared using the Kaplan–Meier method, with the latter group having a significantly shorter liver-related death-free survival (*p* < 0.0001). HCC-free survival was also examined, with a significantly higher incidence of HCC in the severe fibrosis group (*p* = 0.0191) (Figure 2a,b).

In cohort 2 (Figure 1), which was set up to include 90 of the 198 patients included in cohort 1, excluding 108 patients whose serum zinc concentration data were missing at the time of histological diagnosis of AIH, no significant differences in age or sex ratio were found between the mild fibrosis group (n = 63) and the severe fibrosis group (n = 27) (Table 1). 

In the blood test parameters, aspartate transferase (AST) (*p* = 0.013), ALT (*p* = 0.006), albumin (*p* = 0.011), and platelet count (*p* < 0.001) were all significantly lower in the severe fibrosis group, whereas IgG levels were significantly higher (*p* = 0.007). Serum zinc levels were lower, whereas serum ferritin levels were higher (*p* = 0.061, *p* = 0.063, respectively). When liver-related death-free survival was compared for both groups using the Kaplan–Meier method, liver-related death-free survival was significantly shorter in the severe fibrosis group (*p* = 0.0071) (Figure 2c). HCC-free survival was also examined; however, no significant difference was found between the two groups (*p* = 0.0927) (Figure 2d).

### 3.2. Relationship between Blood Inorganic Salt Balance and Liver Fibrosis Progression in Patients with AIH

**Figure 3:**
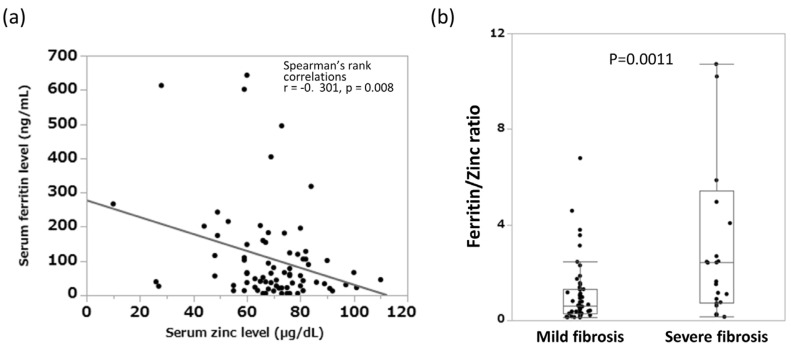
Relationship between blood inorganic salt balance and liver fibrosis progression in patients with AIH. (**a**) Relations between serum zinc and ferritin concentration in patients with autoimmune hepatitis. (**b**) Serum ferritin–zinc ratio classified by liver fibrosis progression. AIH, autoimmune hepatitis.

In cohort 2, we examined the correlation between serum zinc and serum ferritin levels and found a significant inverse correlation between the two (r = −0.301, *p* = 0.008) (Figure 3a). Moreover, in a comparison of the serum ferritin–zinc ratio between the two groups classified according to histological fibrosis progression, the median values for the mild and severe fibrosis groups were 0.62 and 2.41, respectively, with the latter group having significantly higher values (*p* = 0.0011) (Figure 3b).

### 3.3. Measurement and Intercomparison of Various Serum Biomarkers in Predicting Liver Fibrosis Progression That Is Related to the Long-Term Prognosis of Patients with AIH

**Figure 4:**
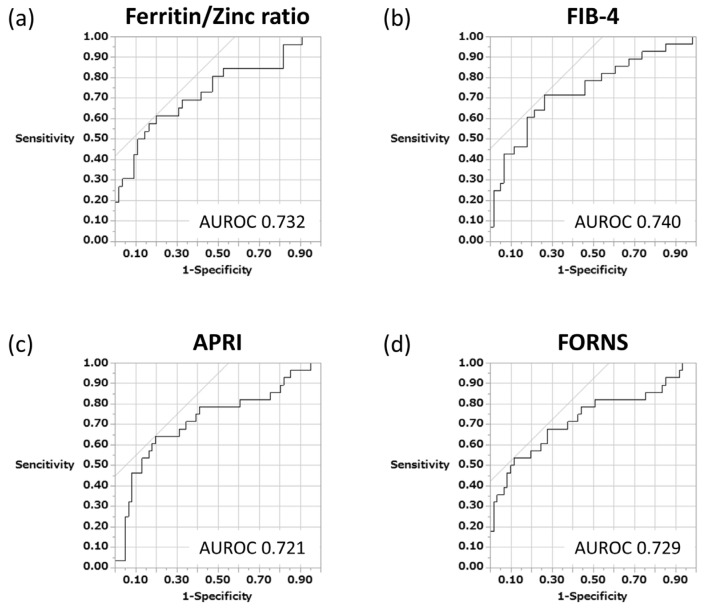
Efficacy of various serum biomarkers in predicting severe hepatic fibrosis of patients with AIH. ROC curve analysis. (**a**) ROC curve for ferritin–zinc ratio (AUC = 0.732, sensitivity = 62%, specificity = 80%). (**b**) ROC curves for FIB-4 (AUC = 0.740, sensitivity = 71%, specificity = 74%). (**c**) ROC curves for APRI (AUC = 0.721, sensitivity = 64%, specificity = 80%). (**d**) ROC curves for Forns index (AUC = 0.729, sensitivity = 54%, specificity = 89%). AIH, autoimmune hepatitis; APRI, aspartate transferase to platelet ratio index; AUC, area under the curve; ROC, receiver operating characteristic.

Studies have already reported the usefulness of serum biomarkers such as FIB-4, APRI, and the Forns index as noninvasive indicators to predict liver fibrosis mainly in chronic hepatitis C and cirrhosis. In this study, in addition to these indices, we examined whether the serum ferritin–zinc ratio, which we originally devised and measured, could be used to accurately distinguish cases of advanced fibrosis in patients with AIH using the ROC analysis method. The results showed that the serum ferritin–zinc concentration ratio (cutoff value 1.5) could successfully extract severe fibrosis cases (AUC 0.732) (Figure 4a). Similarly, FIB-4 (cutoff 2.5), APRI (cutoff 0.7), and the Forns index (cutoff 9.3) were also examined, with AUC values of 0.740, 0.721, and 0.729, respectively. No significant differences in sensitivity and specificity were observed among these four biomarkers when compared with each other (Figure 4b–d).

### 3.4. Ferritin–Zinc Ratio and Long-Term Prognosis of Patients with AIH

**Figure 5:**
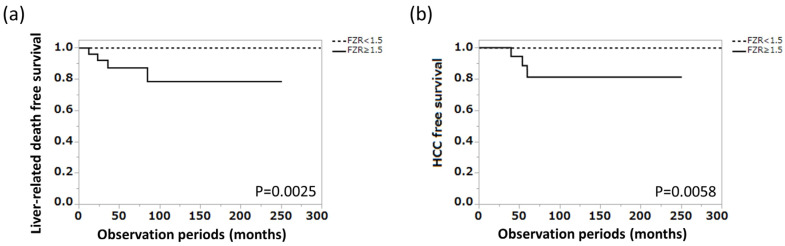
Relations between the level of ferritin–zinc ratio and major clinical outcomes. (**a**) Liver-related death-free survival in cohort 2 (n = 90). (**b**) HCC-free survival in cohort 2 (n = 90). FZR, ferritin–zinc ratio; HCC, hepatocellular carcinoma.

To examine the predictive ability of ferritin–zinc ratio for long-term prognosis in patients with AIH, the patient group in cohort 2 was divided into two different groups based on ferritin–zinc ratio level, and liver-related death-free survival in each group was compared using the Kaplan–Meier method, with the higher group (ferritin–zinc ratio ≥ 1.5) having significantly shorter liver-related death-free survival (*p* = 0.0025). HCC-free survival was also examined, with a significantly higher incidence of HCC in the higher group (*p* = 0.0058) (Figure 5a,b).

## 4. Discussion

Because AIH can have a chronic progressive clinical course, patients have a physical risk of cirrhosis, liver cancer, and ultimately death [24,25]. In this study with an observation period of up to 30 years, we found again that liver cancer incidence and liver-related deaths were significantly higher in patients with advanced liver fibrosis. Recently, in addition to the acute exacerbation type of chronic persistent inflammation, which is a typical case of AIH, an acute hepatitis type has been reported. Despite the clinical phenotype, ultrasound-guided liver biopsy is important to correctly identify the pathology of AIH, including the degree of liver fibrosis progression [26]. Thus, the current EASL and AASLD guidelines state the very important and essential position of liver histological evaluation in AIH diagnosis [27,28]. On the contrary, liver biopsy is invasive, obtaining the patient’s consent for it is difficult, and it requires hospitalization, resulting in high test costs. In addition, the heterogeneity of fibrosis within the liver could lead to sampling errors and a milder assessment of fibrosis progression than is actually the case [29,30]. Therefore, the World Health Organization recommends the use of serological indices as a noninvasive test method to detect liver fibrosis progression in resource-constrained settings [31].

Among serum biomarkers, APRI and FIB-4 are the most widely used for chronic liver diseases such as nonalcoholic liver disease (NAFLD) and viral hepatitis including HBV and HCV [32,33,34], and meta-analyses have also reported about AIH [35,36]. These two serum indicators are considered versatile because of their good reproducibility and high applicability, and they do not incur additional measurement costs [31,37]. However, because these indices require platelet counts for their calculations, they may overestimate fibrosis progression in patients on drugs that can affect platelet counts, such as azathioprine and 6-melcaptopurine. The serum ferritin–zinc ratio that we proposed for the first time does not require a platelet count, and the AUROC was 0.73 in the ROC analysis performed to clarify the ability to differentiate mild from severe fibrosis, which was comparable with that of FIB-4 (AUROC 0.74) and APRI (0.72). In a meta-analysis reported in 2022 by Dong et al. based on 13 original papers and incorporating >2000 patients with AIH, the AUROC values of FIB-4 and APRI for differentiating the mild and severe fibroses were 0.73 and 0.71, respectively [35], which is comparable to our results and may support the reliability of our study. Although most models to predict liver fibrosis including FIB-4 and APRI are complicated and separate formulas are necessary for predicting significant fibrosis, the serum ferritin–zinc ratio is easy to understand and versatile because only simple division is required. Recently, Yuan et al. reported the usefulness of the lymphocyte-to-platelet ratio and immunoglobulin-to-platelet ratio as noninvasive diagnostic tools to assess liver fibrosis progression in patients with untreated AIH and found these indices superior to FIB-4 and APRI in detecting cirrhotic cases [38]. However, future follow-up evaluation is desirable, given the limited number of AIH cases in this study.

Unlike the numerous biomarkers discussed above, the serum ferritin–zinc ratio, which is not affected by the platelet count, is a novel and potentially promising predictor of liver fibrosis in patients with AIH. Iron promotes the conversion of collagen peptides into procollagen and is a potent source of reactive oxygen species in the liver [39], causing hepatocyte injury and DNA damage via increased superoxide and hydroxyl radicals, resulting in hepatitis progression and hepatocarcinogenesis [40]. Ferritin is the protein that regulates excess serum iron, and its level is elevated in the presence of tissue destruction due to chronic inflammation, which promotes biosynthesis and secretion in the reticuloendothelial system [39]. Conversely, serum zinc levels are often low in patients with hepatic disease due to diminished oral intake due to appetite loss or excessive weight loss associated with diuretic use. Zinc inhibits collagen cross-linking and promotes the degradation of cross-linked collagen in collagen metabolism [41]. Thus, the combination of ferritin and zinc, which have opposite directions in the development and inhibition of fibrosis, should be widely investigated in viral liver disease, alcoholic liver disease, and NAFLD.

This study has several limitations that should be considered when interpreting the results. First, this study was performed retrospectively in a university hospital, and the results may be biased by measured or unmeasured confounding factors. Second, the sample size was not large because the prevalence of AIH was not high. Third, the cross-sectional analysis failed to examine the direct relationship between the new biomarker and liver fibrosis progression during individual patient follow-up. Fourth, the conclusions would be preferably confirmed in a prospective study with a larger sample size because this study lacks a validation cohort. However, regardless of the aforementioned limitations, we believe that additional molecular or statistical evidence could further strengthen these results and the ferritin–zinc ratio proposed in this study would be a useful and simple noninvasive tool that is available to efficiently identify the population with severe liver fibrosis among patients with AIH. Since this index can be used to estimate the degree of liver fibrosis progression in patients with AIH and can be applied to predict the long-term prognosis of patients, it would be worthwhile to undertake assessment of this measurement as a multicenter prospective study in the future.

In conclusion, the serum ferritin–zinc ratio proposed in this study was identified as an independent factor associated with liver fibrosis progression in patients with AIH and may be a useful tool for monitoring liver fibrosis progression.

## Figures and Tables

**Table 1:** Clinical profiles of patients with autoimmune hepatitis in this study.

Observational Study	Cohort 1	Cohort 2			
	Total Patients (n = 198)	Total Patients (n = 90)	Mild Fibrosis (n = 63)	Severe Fibrosis (n = 27)	*p*
Age (years)	62.0 [51.0–69.0]	62.5 [52.8–67.3]	60.0 [52.0–67.0]	64.5 [54.8–70.3]	0.270
Sex (male/female)	33/165	16/74	8/55	7/20	0.135
Fibrosis (F1/F2/F3/F4)	76/49/40/33	39/24/18/9	39/24/0/0	0/0/18/9	>0.001
AST (U/L)	87.0 [48.8–259.8]	83.5 [52.0–234.0]	82.5 [54.0–359.5]	84.0 [45.3–122.0]	0.013
ALT (U/L)	81.5 [46.0–323.8]	86.5 [52.8–229.8]	98.5 [62.3–329.8]	70.5 [48.3–103.3]	0.006
Gamma GT (U/L)	111.5 [57.0–210.0]	128.0 [62.0–296.8]	128.0 [62.3–296.8]	126.5 [60.0–298.3]	0.897
Total bilirubin (mg/dL)	1.1 [0.7–2.3]	1.0 [0.7–2.0]	1.0 [0.7–1.4]	1.2 [1.0–2.1]	0.079
Albumin (g/dL)	4.0 [3.6–4.3]	4.1 [3.8–4.3]	4.1 [3.9–4.3]	3.9 [3.2–4.3]	0.011
Total cholesterol (mg/dL)	184.0 [154.0–214.0]	192.5 [160.0–222.8]	194.0 [162.5–223.8]	176.0 [134.0–208.3]	0.437
Ferritin (ng/mL)	161.4 [80.8–351.7]	243.0 [93.0–314.5]	128.4 [64.2–301.9]	295.3 [255.8–437.1]	0.063
Zinc (µg/dL)	69.0 [60.0–79.8]	69.0 [60.0–79.8]	73.0 [64.0–81.8]	63.0 [53.0–71.8]	0.061
IgG (mg/dL)	2090.0 [1600.5–2658.5]	2035.0 [1533.5–2564.5]	1825.5 [1472.3–2375.3]	2167.4 [1940.3–3258.0]	0.007
Platelets (×10^4^/µL)	18.5 [12.4–23.4]	19.0 [13.1–23.9]	21.3 [17.8–25.0]	13.2 [10.5–16.3]	>0.001

AST, aspartate transferase; ALT, alanine aminotransferase.

## Data Availability

All the data used to support the findings of this study are included within the article.

## Abbreviations

AIH, autoimmune hepatitis; APRI, aspartate transferase to platelet ratio index; AUROC, area under the receiver operating characteristic curve; FZR, ferritin–zinc ratio; HCC, hepatocellular carcinoma.
